# Recruitment Maneuver to Reduce Postoperative Pulmonary Complications after Laparoscopic Abdominal Surgery: A Systematic Review and Meta-Analysis

**DOI:** 10.3390/jcm11195841

**Published:** 2022-10-01

**Authors:** Shuaijie Pei, Wei Wei, Kai Yang, Yiyi Yang, Yu Pan, Jinrui Wei, Shanglong Yao, Haifa Xia

**Affiliations:** 1 Department of Anesthesiology, Union Hospital, Tongji Medical College, Huazhong University of Science and Technology, Wuhan 430022, China; 2 Department of Anesthesiology, The People’s Hospital of Honghu, Honghu 433200, China; 3 Department of Critical Care Medicine, The People’s Hospital of Honghu, Honghu 433200, China

**Keywords:** recruitment maneuver, mechanical ventilation, laparoscopic, postoperative pulmonary complication, meta-analysis

## Abstract

Background: Lung-protective ventilation strategies are recommended for patients undergoing mechanical ventilation. However, there are currently no guidelines to follow regarding recruitment maneuvers (RMs). We attempted to identify the effects of RMs on patients undergoing laparoscopic abdominal surgery. Methods: We searched for randomized controlled trials (RCTs) in PubMed, the Cochrane Library databases, Embase, Web of Science and the ClinicalTrials.gov registry for trials published up to December 2021. The primary outcome was postoperative pulmonary complications (PPCs). The secondary outcomes consisted of the static lung compliance, driving pressure (DP), intraoperative oxygenation index (OI), OI in the post-anesthesia care unit (PACU), mean arterial pressure (MAP) and heart rate (HR). Seventeen RCTs with a total of 3480 patients were examined. Results: Patients who received RMs showed a considerable reduction in PPCs (risk ratio (RR) = 0.70; 95% confidence interval (CI): 0.62 to 0.79; *p* < 0.01), lower DP (weighted mean difference (WMD) = −3.96; 95% CI: −5.97 to −1.95; *p* < 0.01), elevated static lung compliance (WMD = 10.42; 95% CI: 6.13 to 14.71; *p* < 0.01) and improved OI (intraoperative: WMD = 53.54; 95% CI: 21.77 to 85.31; *p* < 0.01; PACU: WMD = 59.40; 95% CI: 39.10 to 79.69; *p* < 0.01) without substantial changes in MAP (WMD = −0.16; 95% CI −1.35 to 1.03; *p* > 0.05) and HR (WMD = −1.10; 95% CI: −2.29 to 0.10; *p* > 0.05). Conclusions: Recruitment maneuvers reduce postoperative pulmonary complications and improve respiratory mechanics and oxygenation in patients undergoing laparoscopic abdominal surgery. More data are needed to elucidate the effect of recruitment maneuver on the circulatory system.

## 1. Introduction

Laparoscopic surgery is becoming more and more common due to its advantages of minimal incisions, clear surgical views and reduced postoperative hospital stays [1,2]. However, the pneumoperitoneum and Trendelenburg position cause cephalad displacement of the diaphragm, which reduces pulmonary compliance and functional residual capacity (FRC) and greatly increases the risk of postoperative pulmonary complications (PPCs) [3]. PPCs have been reported to be associated with increased early postoperative mortality, ICU readmission and length of hospital stay [4,5]. Therefore, it is critical to prevent PPCs in the perioperative period. 

Pulmonary-protective ventilation strategies, including low tidal volume (TV) ventilation, positive end-expiratory pressure (PEEP) ventilation and the recruitment maneuver (RM), are among the beneficial means for reducing PPCs that many researchers have studied [6]. 

There remains controversy and a lack of guidelines to follow regarding the RM. The RM can reverse pulmonary atelectasis to some extent and maintain the alveolar opening by increasing the airway pressure. Depending on the fluctuation of airway pressure, RMs can be divided into the sustained RM and stepwise RM. The stepwise RM comprises a stepwise increase in TV and stepwise increase in PEEP [7]. Previous systematic reviews have reported that the RM in patients undergoing general anesthesia improves oxygenation and reduces PPCs [8]. However, the study did not distinguish between laparoscopic and open surgery, and the number of included publications was limited. Another large multicenter randomized controlled trial (RCT) showed that the open-lung ventilation strategy was not effective in reducing the incidence of PPCs compared to conventional protective ventilation [4]. Therefore, we performed this meta-analysis of RCTs to discuss the effect of the RM on PPCs, the respiratory mechanics and the hemodynamics during laparoscopic abdominal surgery.

## 2. Materials and Methods

We report the results of this meta-analysis in compliance with the preferred reporting items for systematic reviews and meta-analyses (PRISMA) guidelines [9]. This study is registered in the International Prospective Register of Systematic Reviews (PROSPERO) with registration number CRD42022315969.

### 2.1. Search Strategy

PubMed, Embase, the Cochrane Library databases, Web of Science and the ClinicalTrials.gov registry were searched, and we included literature published before December 2021. We used Medical Subject Headings (MeSH) terms and multiple combinations related to “Abdomen”, “Laparoscopy” and “Hand-Assisted Laparoscopy” for retrieval. With no MeSH terms associated with the RM, we used “recruitment maneuver”, “recruitment maneuvers”, “RM”, “open lung”, “protected ventilation” or “protective ventilation” for the search based on previous literature [10,11]. The study type was restricted to RCTs. There were no language restrictions. Finally, the above findings were combined to produce our results. The general search strategy is provided in Table 1.

### 2.2. Selection Criteria

Studies were selected for inclusion based on the following criteria. The screening process was performed independently by SP and WW.

- The subjects were adult patients subjected to laparoscopic abdominal surgery requiring general anesthesia and mechanical ventilation.
- The included studies were required to compare RM groups with non-RM groups (or control groups).
- The included studies had to plainly state the mechanical ventilation strategies, and inclusion and exclusion criteria. Postoperative pulmonary complications had to be reported.
- Studies containing patients who were minors or had previous lung disease were excluded.

### 2.3. Data Extraction

Two researchers (YP and JW) independently collected the following information from the original texts: the first author, publication year, ASA grading, age, gender, sample size, body mass index (BMI), surgery type, ventilation settings (the TV, airway pressure, PEEP and RM), hemodynamic parameters (mean arterial pressure (MAP) and heart rate (HR)), respiratory indicators (the incidence of PPC, static lung compliance, driving pressure (DP), intraoperative oxygenation index (OI) and OI in the post-anesthesia care unit (PACU)). We calculated the OI as the arterial partial pressure of oxygen/the inspiratory oxygen fraction (PaO_2_/FiO_2_). The DP was computed as (airway plateau pressure—PEEP), while the static lung compliance was measured as TV/(airway plateau pressure—PEEP). If the patients were divided into multiple groups in the article, only data from the RM group (followed by PEEP) and conventional ventilation group (without RM) were recorded. Any disputes were adjudicated by SY and HX. 

Continuous data and dichotomous data were expressed as the means ± standard deviations (SDs) and numbers, respectively. If continuous data were provided as medians, interquartile ranges or ranges, we transformed them to means and SDs on the basis of the Cochrane Collaboration recommendations [12].

### 2.4. Statistical Analysis

We used Review Manager 5.3 (Cochrane Collaboration, Oxford, UK) and Stata17.0 (StataCorp, College Station, TX, USA) to aggregate the data in accordance with the PRISMA standards [13]. The inverse-variance and Mantel–Haenszel methods were performed separately to assess continuous and dichotomous variables among merged trials. We calculated the weighted mean differences (WMDs) and 95% confidence intervals (CIs) for continuous variables, while for dichotomous variables, we derived the risk ratios (RRs) and 95% CIs. 

The heterogeneity was assessed using Cochrane’s Q test. *p* > 0.10 indicated that heterogeneity was not detected, and the fixed-effects model was used to calculate the combined statistics. Additionally, *p* < 0.10 suggested significant heterogeneity, and the random-effects model was chosen. The statistic I^2^ was used to quantify the magnitude of the heterogeneity. I^2^ ranges from 0% to 100%. A negative value of I^2^ was the same as 0. I^2^ = 0 means no detected heterogeneity, and a larger number denoted increased heterogeneity. According to the Higgins classification, I^2^ values of 25%, 50% and 75% represented low, moderate and high heterogeneity, respectively [14]. A heterogeneity of no more than 50% was generally considered acceptable. Sensitivity analysis, which excluded some literature, was used to identify studies with high heterogeneity. Subgroup analysis was performed according to the BMI, age, number of RMs, type of RMs, recruited pressure and PEEP. Egger’s test and Begg’s test were applied to evaluate the publication bias. 

### 2.5. Quality Assessment

Two researchers (KY and YY) judged the methodological quality utilizing the Cochrane Collaboration technique, including random sequence generation, allocation concealment, performance bias, outcome blinding bias, incomplete data bias, selective reporting bias and others. Each item comprised three degrees of bias risk: low risk of bias, unclear risk of bias and high risk of bias. We also used GRADEpro (McMaster University, Hamilton, ON, 2014) [15], a method for assessing the quality of evidence and degree of suggestions for strategies, to appraise the quality of evidence.

## 3. Results

### 3.1. Study Characteristics

**Figure 1:**
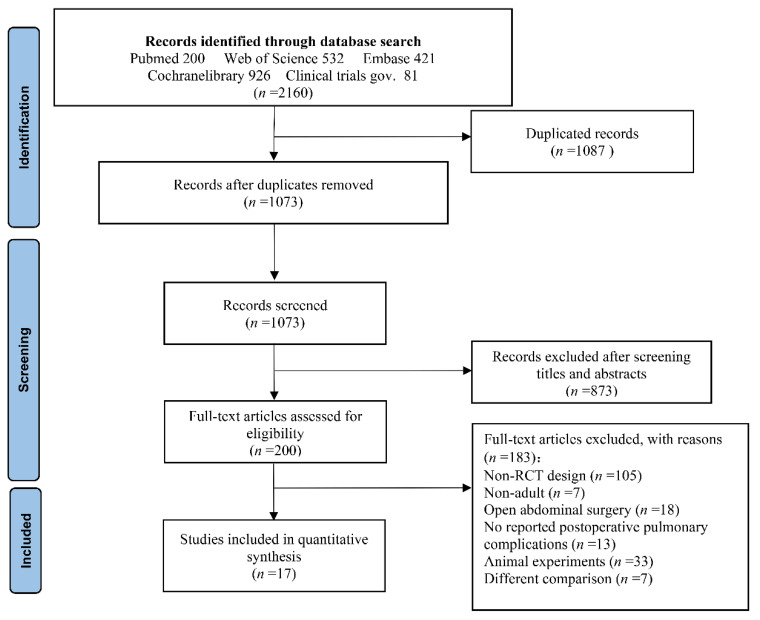
Flow chart of study screening.

The study-screening process is shown in Figure 1. Initially, we retrieved 2160 likely correlated studies (Pubmed, 200; Web of Science, 532; Embase, 421; Cochrane Library database, 926; Clinicaltrials.gov registry, 81). Then, 1087 duplicates were removed. The remaining literature’s titles and abstracts were thoroughly reviewed, and 873 irrelevant publications were eliminated. Finally, 200 full-text articles were screened, and only 17 of them matched the inclusion requirements. The reasons for the 183 exclusions were as follows: 105 were non-RCTs, 7 were non-adult studies, 18 reported open abdominal surgery, 13 did not report PPCs, 33 were animal experiments and 7 did not compare recruitment maneuvers to conventional mechanical ventilation. Finally, 17 RCTs with a total of 3480 patients were examined [16,17,18,19,20,21,22,23,24,25,26,27,28,29,30,31,32]. The details of the included studies are presented in Table 2.

**Figure 2:**
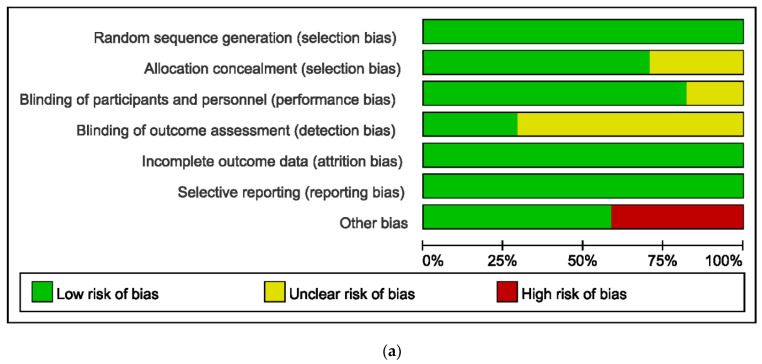

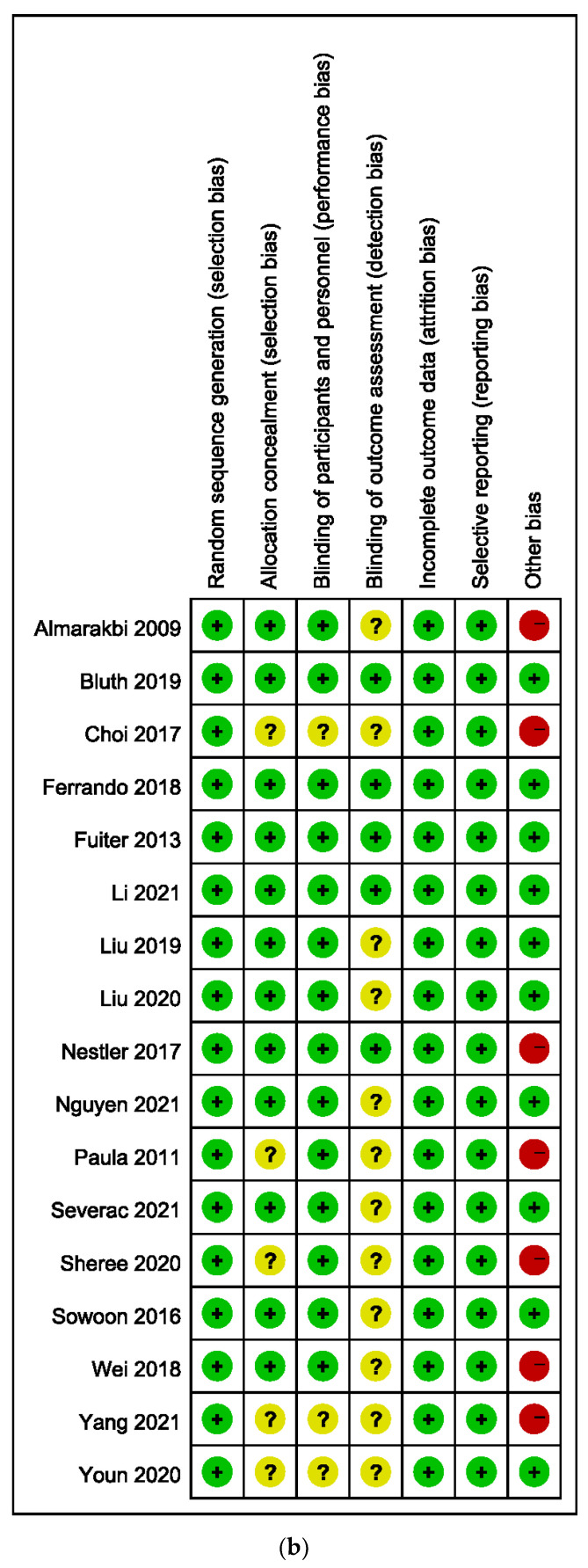
Evaluation of risk bias for included RCTs: (**a**) percentage plot of seven types of bias for the included studies; (**b**) summary of bias for each study [16,17,18,19,20,21,22,23,24,25,26,27,28,29,30,31,32].

Figure 2 illustrates the results of the quality evaluation with Review Manager 5.3. All the studies presented low risk in terms of random sequence generation. Due to the completeness of the outcome data and lack of selective reporting, the risks of attrition bias and reporting bias were likewise evaluated as low. Five trials did not provide information on allocation concealment [18,26,28,31,32]. Twelve studies did not report the blinding of the outcome evaluators [15,18,22,23,25,26,27,28,29,30,31,32]. Seven studies showed a high risk of “other bias” owing to the small sample sizes [16,18,24,26,28,30,31]. We did not detect publication bias for each index using Begg’s test and Egger’s test with Stata (version 17.0, StataCorp, College Station, TX, USA).

### 3.2. Grading Evidence Quality 

The results of the assessment of the evidence quality using GRADEpro are presented in Table 3. Based on the risk of bias, inconsistency, indirectness, imprecision and publication bias, we classified the evidence quality into four levels: high, moderate, low and very low. In terms of the risk of bias, we ranked the risk for all 18 indicators assessed as not serious. The inconsistency for the static lung compliance, driving pressure, intraoperative OI and OI in the PACU was rated as severe due to I^2^ > 50%, which indicates unacceptable heterogeneity. The indirectness and imprecision for all the indicators were classified as not serious because all the studies made direct comparisons between RMs and control groups with adequate sample sizes. No publication bias was found according to Egger’s test and Begg’s test. Due to the RR being less than 0.5, the quality of evidence for single RMs, sustained RMs, recruited pressure < 40 and comparisons to ZEEP was improved. Finally, we had moderate confidence in the outcomes for the static lung compliance, driving pressure, intraoperative OI and OI in the PACU, while we had high confidence in the rest of the results.

### 3.3. Primary Outcomes

#### 3.3.1. Incidence of PPC

**Figure 3:**
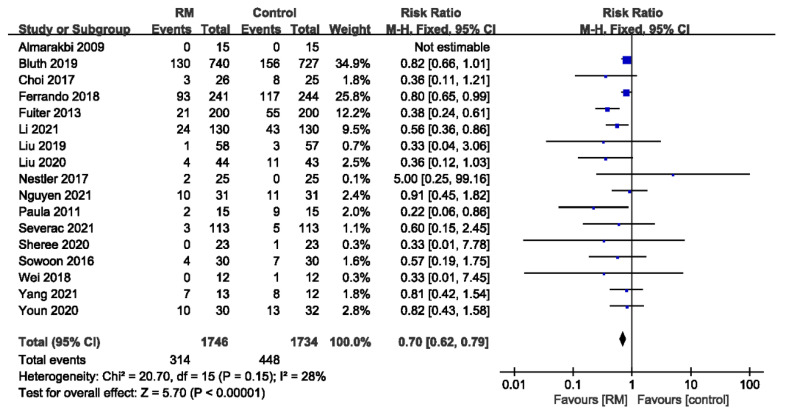
Forest plot for the incidence of PPCs between RM and control groups [16,17,18,19,20,21,22,23,24,25,26,27,28,29,30,31,32].

Seventeen studies with a total of 3480 patients reported PPCs whose general incidence was about 21.9% (448/1734 in the non-RM group and 314/1746 in the RM group). RMs significantly reduced PPCs, with low heterogeneity, compared to the control group (RR = 0.70; 95% CI: 0.62 to 0.79; *p* < 0.01; *p* for heterogeneity > 0.10; I^2^ = 28%) (Figure 3).

#### 3.3.2. Subgroup Analysis of PPC by BMI

**Figure 4:**
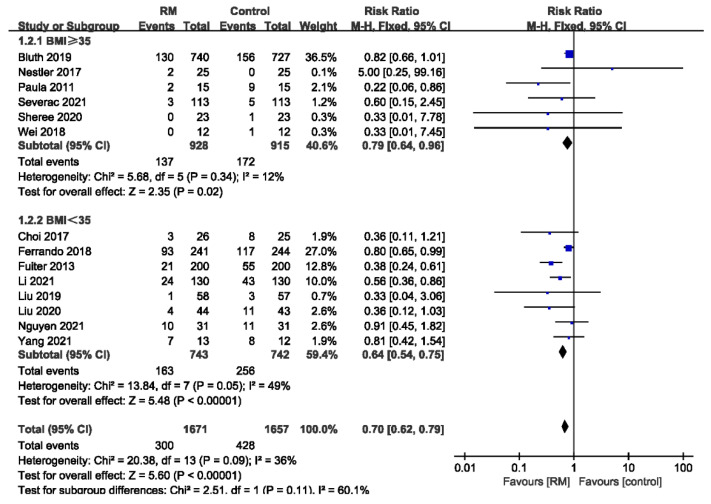
Forest plot for subgroup analysis of the incidence of PPCs between RM and control groups. Grouped by BMI: BMI ≥ 35 kg/m^2^, BMI < 35 kg/m^2^ [17,18,19,20,21,22,23,24,25,26,27,28,30,31].

Six studies reported the incidence of PPCs in obese patients (BMI ≥ 35 kg/m^2^), while eight studies reported non-obese patients (BMI < 35 kg/m^2^). RMs reduced the incidence of PPCs in obese and non-obese patients with acceptable heterogeneity (BMI ≥ 35: RR= 0.79; 95% CI: 0.64 to 0.96; *p* < 0.05; *p* for heterogeneity > 0.10; I^2^ = 12%; BMI < 35: RR = 0.64; 95% CI: 0.54 to 0.75; *p* < 0.01; *p* for heterogeneity = 0.05; I^2^ = 49%) (Figure 4).

#### 3.3.3. Subgroup Analysis of PPCs by Age

**Figure 5:**
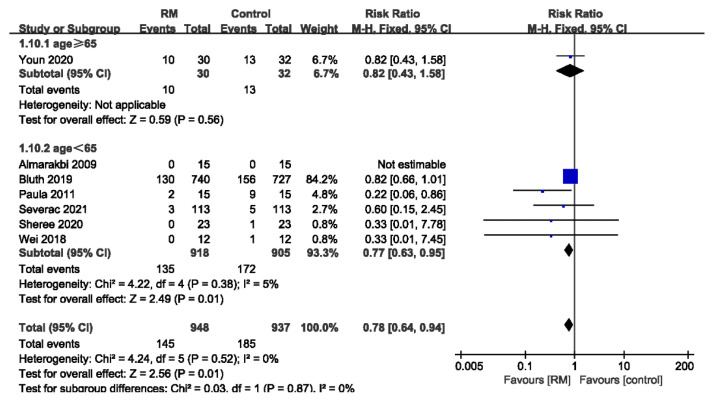
Forest plot for subgroup analysis of the incidence of PPCs between RM and control groups. Grouped by age: age ≥ 65 years, age < 65 years [16,17,26,27,28,30,32].

Only one study included subjects who were all elderly patients (age ≥ 65 years). Six studies, with a total of 1823 patients, included non-elderly subjects (age < 65 years). The results showed that RMs reduced the incidence of PPCs in non-elderly patients but were not effective in elderly patients (age ≥ 65: RR= 0.82; 95% CI: 0.43 to 1.58; age < 65: RR= 0.77; 95% CI: 0.63 to 0.95; *p* < 0.05; *p* for heterogeneity > 0.10; I^2^ = 5%). We should be cautious regarding the effect of RMs on the elderly due to the insufficient number of studies (Figure 5).

#### 3.3.4. Subgroup Analysis of PPCs by the Number of RMs

**Figure 6:**
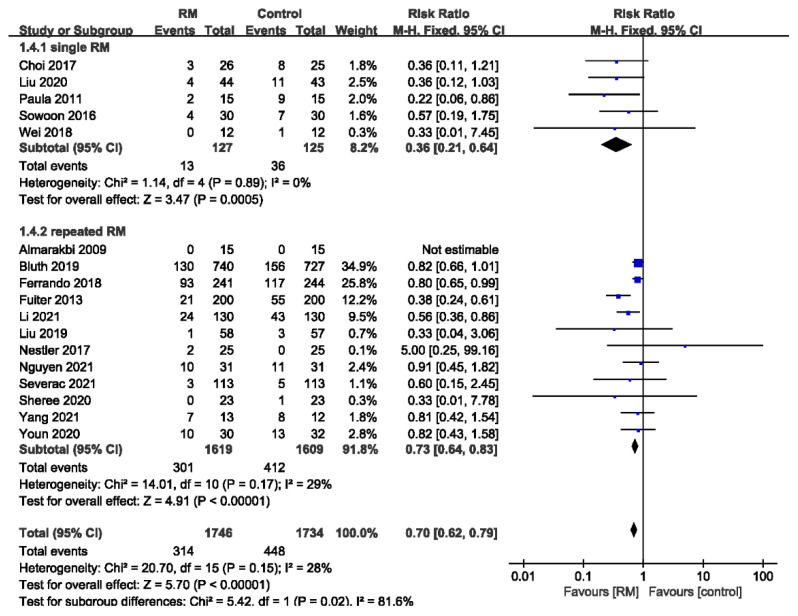
Forest plot for subgroup analysis of the incidence of PPCs between RM and control groups. Grouped by the number of RM: single RM, repeated RM [16,17,18,19,20,21,22,23,24,25,26,27,28,29,30,31,32].

Five studies used single RMs during the procedures. The remaining 12 studies used repeated RMs. The results showed that a single RM significantly reduced the incidence of PPCs with no heterogeneity (RR = 0.36; 95% CI 0.21 to 0.64; *p* < 0.01; *p* for heterogeneity > 0.10; I^2^ = 0%). The use of repeated RMs also reduced the incidence of PPCs with acceptable heterogeneity (RR = 0.73; 95% CI: 0.64 to 0.83; *p* < 0.01; *p* for heterogeneity > 0.10; I^2^ = 29%). A single RM may be more efficient than repeated RMs (*p* for heterogeneity < 0.05; I^2^ = 81.6%) (Figure 6).

#### 3.3.5. Subgroup Analysis of PPCs by the Type of RM

**Figure 7:**
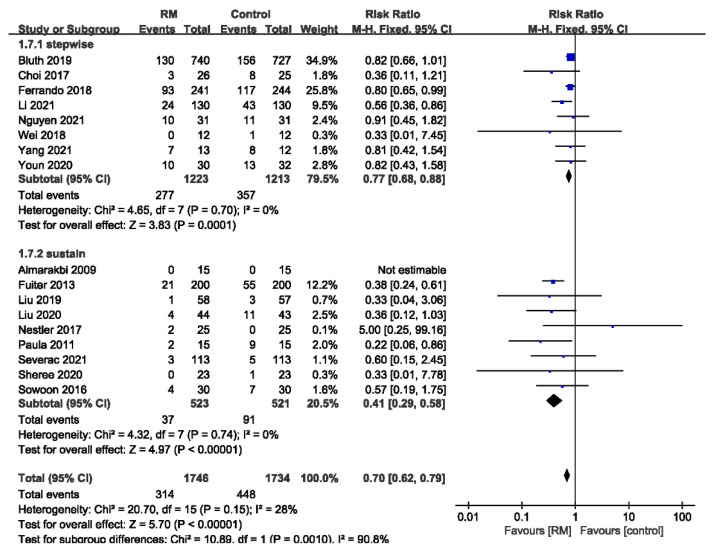
Forest plot for subgroup analysis of the incidence of PPCs between RM and control groups. Grouped by the type of RM: stepwise RM, sustained RM [16,17,18,19,20,21,22,23,24,25,26,27,28,29,30,31,32].

Eight studies used stepwise RMs, while the other nine used sustained RMs. The results showed that stepwise RMs reduced the incidence of PPCs and sustained RMs achieved this effect more significantly. No heterogeneity was detected in either subgroup (stepwise: RR= 0.77; 95% CI: 0.68 to 0.88; *p* < 0.01; *p* for heterogeneity > 0.10; I^2^ = 0%; sustained: RR = 0.41; 95% CI: 0.29 to 0.58; *p* < 0.01; *p* for heterogeneity > 0.10; I^2^ = 0%; *p* for subgroup differences < 0.01) (Figure 7).

#### 3.3.6. Subgroup Analysis of PPCs by Recruited Pressure

**Figure 8:**
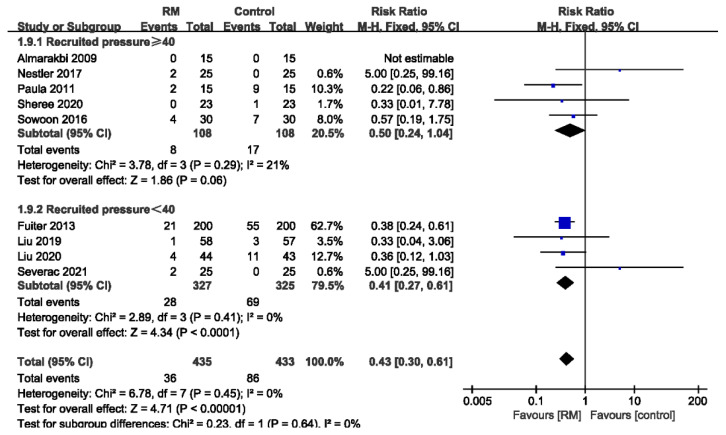
Forest plot for subgroup analysis of the incidence of PPCs between RM and control groups. Grouped by recruited pressure: recruited pressure ≥ 40 cm H_2_O, recruited pressure < 40 cm H_2_O [16,20,22,23,24,26,27,28,29].

Rothen et al. [33] reported that a recruited pressure greater than 40 cm H_2_O is required to ensure opening in pulmonary atelectasis. In Figure 8, we divide the included studies into two groups according to the recruited pressure at 40 cm H_2_O. There are five studies with recruited pressures ≥ 40 cm H_2_O, while four studies had recruited pressures < 40 cm H_2_O. The results showed that the incidence of PPCs was reduced when the recruited pressure was less than 40 cm H_2_O, while a recruited pressure ≥ 40 cm H_2_O was not beneficial for improving outcomes (recruited pressure ≥ 40 cm H_2_O: RR = 0.50; 95% CI: 0.24 to 1.04; *p* > 0.05; *p* for heterogeneity > 0.10; I^2^ = 21%; recruited pressure < 40 cm H_2_O: RR= 0.41; 95% CI: 0.27 to 0.61; *p* < 0.01; *p* for heterogeneity > 0.10; I^2^ = 0%). The heterogeneity was reduced to 0% in sensitivity analysis by excluding the study of Nestler et al. [24] from the subgroup with recruited pressures ≥ 40 cm H_2_O (RR = 0.37; 95% CI: 0.16 to 0.84; *p* < 0.05; *p* for heterogeneity > 0.10; I^2^ = 0%). 

#### 3.3.7. Subgroup Analysis of PPCs by ZEEP or PEEP Used in Control Group

**Figure 9:**
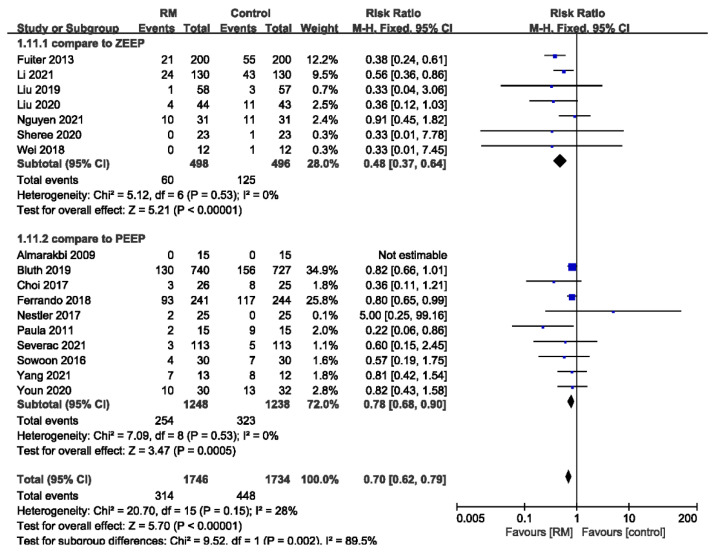
Forest plot for subgroup analysis of the incidence of PPCs between RM and control groups. Grouped by ZEEP or PEEP used in control group: compare to ZEEP, compare to PEEP [16,17,18,19,20,21,22,23,24,25,26,27,28,29,30,31,32].

In the open-lung strategy, the RM is usually used in combination with PEEP. Some control groups of the included studies used PEEP, while the rest used zero end-expiratory pressure (ZEEP). We performed subgroup analysis based on whether PEEP was used in the control group. The results showed that there was a significant difference in the incidence of PPCs, with no heterogeneity, regardless of whether PEEP was used in the control group (compared to ZEEP: RR = 0.48; 95% CI: 0.37 to 0.64; *p* < 0.01 *p* for heterogeneity > 0.10; I^2^ = 0%; compared to PEEP: RR = 0.78; 95% CI: 0.68 to 0.90; *p* < 0.01; *p* for heterogeneity > 0.10; I^2^ = 0%). The protective effect in comparison with ZEEP was more pronounced (*p* for subgroup differences < 0.01) (Figure 9).

### 3.4. Secondary Outcomes 

#### 3.4.1. Static Lung Compliance 

**Figure 10:**
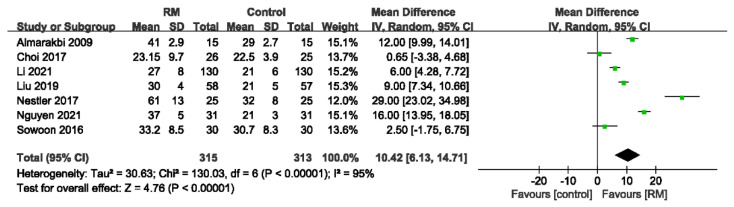
Forest plot for static lung compliance between RM and control groups [16,18,21,22,24,25,29].

Seven studies involving a total of 628 patients reported static lung compliance, and the data suggest that the RM is beneficial in enhancing lung compliance but is highly heterogeneous (WMD: 10.42; 95% CI: 6.13 to 14.71; *p* < 0.01; *p* for heterogeneity < 0.10; I^2^ = 95%) (Figure 10).

#### 3.4.2. Driving Pressure

**Figure 11:**
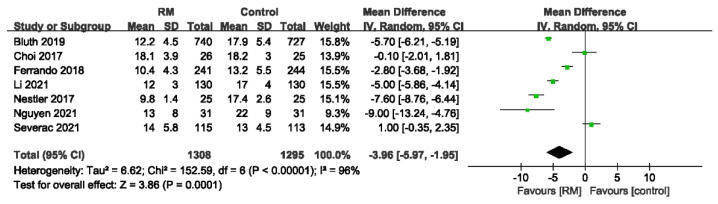
Forest plot for driving pressure between RM and control groups [17,18,19,21,24,25,27].

The driving pressure was reported in seven trials with a total of 2603 individuals, and the findings showed that the RM was useful in reducing the DP but was very heterogeneous (WMD: −3.96; 95% CI: −5.97 to −1.95; *p* < 0.01; *p* for heterogeneity < 0.10; I^2^ = 96%) (Figure 11).

#### 3.4.3. Intraoperative Oxygenation Index 

**Figure 12:**
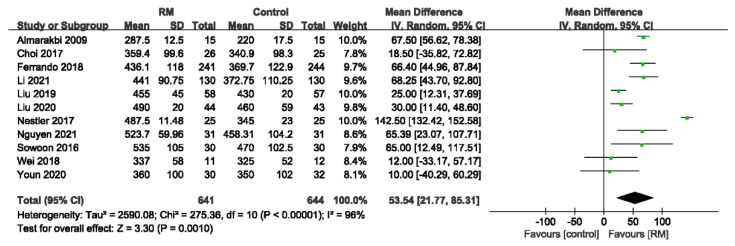
Forest plot for intraoperative OI between RM and control groups [16,18,19,21,22,23,24,25,29,30,32].

The intraoperative OIs were reported for 1285 patients from 11 studies. The global data suggested that the RM could improve the intraoperative OI but with high heterogeneity (WMD: 53.54; 95% CI: 21.77 to 85.31; *p* < 0.01, *p* for heterogeneity < 0.10; I^2^ = 96%) (Figure 12).

#### 3.4.4. Oxygenation Index in Post-Anesthesia Care Unit

**Figure 13:**
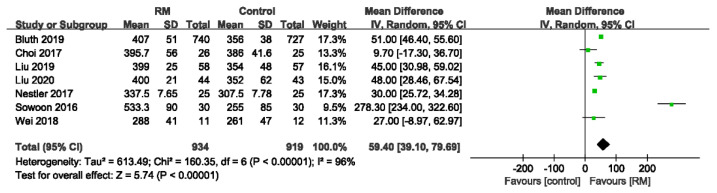
Forest plot for OI in PACU between RM and control groups [17,18,22,23,24,29,30].

Seven studies examined the postoperative OIs in patients who underwent laparoscopic abdominal surgery. The RM group had higher OIs than the control group (WMD: 59.40; 95% CI: 39.10 to 79.69; *p* < 0.05; *p* for heterogeneity < 0.10; I^2^ = 96%) (Figure 13).

#### 3.4.5. Mean Arterial Pressure

**Figure 14:**
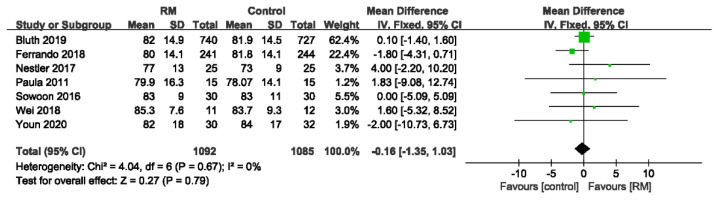
Forest plot for mean arterial pressure between RM and control groups [17,19,24,26,29,30,32].

The MAPs were reported in seven studies involving 2177 patients. The MAP was not significantly different between the conventional mechanical ventilation and RM groups. No heterogeneity was observed in the results (WMD: −0.16; 95% CI: −1.35 to 1.03; *p* > 0.05; *p* for heterogeneity > 0.10; I^2^ = 0%) (Figure 14).

#### 3.4.6. Heart Rate

**Figure 15:**
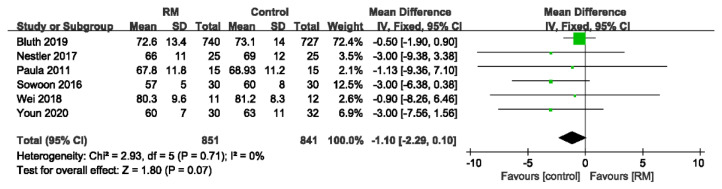
Forest plot for subgroup analysis of heart rate between RM and control groups [17,24,26,29,30,32].

Six studies, with a total of 1692 patients, reported HR. Overall, there was no significant difference in the effect of the RM on HR compared to control (WMD: −1.10; 95% CI: −2.29 to 0.10; *p* > 0.05; *p* for heterogeneity > 0.10; I^2^ = 0%) (Figure 15). 

## 4. Discussion

This meta-analysis included 17 RCTs comparing RMs and conventional mechanical ventilation in patients undergoing laparoscopic abdominal surgery. The types of procedures included robot-assisted laparoscopic radical prostatectomy (RARP), laparoscopic colorectal cancer resection, laparoscopic gastric cancer radical surgery, laparoscopic total hysterectomy and laparoscopic bariatric surgery. Patients undergoing laparoscopic abdominal surgery are at high risk for PPCs. The RM is an effective method for improving pulmonary atelectasis. However, there are few systematic reviews or meta-analyses reporting the effects of RMs on patients undergoing laparoscopic abdominal surgery. Therefore, a comprehensive analysis of previous RCTs was necessary. Our results showed that, for patients undergoing laparoscopic abdominal surgery, RMs reduced the incidence of PPCs and the driving pressure and improved the oxygenation and static lung compliance compared with controls, without significant differences in the MAP and HR. The heterogeneity was higher for the static lung compliance, DP, intraoperative OI and OI in the PACU, while less heterogeneity was found for PPCs, the MAP and the HR. Heterogeneity may arise from several sources. First, the enrolled patients had a wide age range and underwent different laparoscopic abdominal procedures. Second, the intraoperative ventilation strategy is highly variable. The tidal volume, RM and PEEP can affect the oxygenation and respiratory mechanics. Third, the DP and OI are directly provided in some articles. For studies where the data are not available, we calculated them using equations.

Obese patients are likely to undergo laparoscopic bariatric surgery; they usually have reduced functional residual capacity (FRC), impaired oxygen reserves and comorbidities [34,35]. Pulmonary atelectasis, which plays an important role in PPCs [36], is further aggravated under the influence of general anesthesia, pneumoperitoneum and the Trendelenburg position. The role of RMs in obese patients is still worth discussing. Several studies have demonstrated that RMs can ameliorate PPCs. Reinius et al. [37] concluded that RMs alone were not sufficient to maintain improved respiratory function. We performed subgroup analysis based on BMI and found that RMs reduced PPCs in both obese and non-obese patients, with no significant difference between the two subgroups. This was contrary to the finding of Cui et al. [38], whose meta-analysis indicated that RMs did not improve PPCs in obese patients. However, the fact that there were only two studies with a total of 70 patients in the obese group and high heterogeneity lent low credibility to their findings.

The majority of patients undergoing laparoscopic radical prostatectomy and tumor resection are elderly. With increasing age, elderly patients have compromised respiratory compliance, increased closing volumes and impaired airway protective reflexes. These changes make them more prone to abnormal gas exchange and pulmonary atelectasis. Our subgroup analysis based on age showed that RMs reduced the incidence of PPCs in non-elderly patients but were not effective in elderly patients. However, there was only one study in the elderly group, containing 62 patients, which made the results less reliable. The meta-analysis by Cui et al. [38] showed that RMs reduced the incidence of PPCs in elderly patients undergoing general anesthesia. However, Cui et al. classified patients as elderly or non-elderly based on age 60, whereas our study used 65 as the cut-off. Furthermore, the research of Cui et al. included both spinal fusion surgery and open surgery, while our analysis only included laparoscopic abdominal surgery. These variations all reduced the comparability of two investigations. More high-quality studies are needed to validate the effect of RMs on PPCs in elderly individuals.

In addition to patient characteristics, the RM itself is worthy of further discussion. Some experiments used single RM [18,23,26,29,30], while others employed repeated RMs [16,17,19,20,21,22,24,25,27,28]. The results showed that both methods reduced PPCs. Unexpectedly, single RM had even lower risk ratios and a statistically significant difference compared to the other subgroup, which indicated a more pronounced effect. Although the RM is considered to be an effective means of reducing pulmonary atelectasis and preventing PPCs, repeated RMs are accompanied by an increased risk of lung hyperinflation and hemodynamic instability in normal lungs. The single RM in the included studies was administered after intubation or pneumoperitoneum, a phase with a higher incidence of pulmonary atelectasis and greater risk of the development of hemodynamic instability due to medications, positive pressure ventilation and pneumoperitoneum. There is no high-quality evidence to recommend routine RMs after tracheal intubation for patients undergoing general anesthesia, and anesthesiologists need to assess the patient’s risk–benefit ratio to tailor treatment. Continuous hemodynamic and SpO2 monitoring is necessary during RMs.

RMs are usually classified as sustained RMs and stepwise RMs. A sustained RM involves setting the airway pressure at a high value and ventilating continuously for a period of time. This is commonly achieved by adjusting the airway-pressure-limiting valve on the ventilator and squeezing the air reservoir. The sustained RM is easy to perform and is widely used in clinical settings. However, when switching back to machine-controlled mode, there is a risk that the alveoli will re-collapse. A stepwise RM gradually boosts the airway pressure by stepping up the tidal volume or PEEP. The stepwise RM is ventilator driven and can be followed by PEEP titration. The operation is complicated and time consuming. As shown in Figure 7, subgroup analysis showed that the risk ratio was lower in the sustained RM group, and the difference was considered statistically significant compared to the stepwise RM group. No heterogeneity was found in either subgroup. This is consistent with the findings of Cui et al. The incidence of PPC in patients receiving stepwise RM was 22.6% while that in patients receiving sustained RM was 7.1%. Significantly, the included studies only compared the RM and control groups. No direct comparison of different RMs was performed. From the available data, we could not identify which RM was more effective. A study by Rothen et al. [33] based on CT imaging suggested that a recruited pressure of 40 cm H_2_O was efficient in reversing pulmonary atelectasis. We performed further subgroup analysis accordingly. Our results showed that the subgroup with a recruited pressure greater than 40 cm H_2_O showed a poor reduction in the incidence of PPCs, while that with a recruited pressure less than 40 cm H_2_O was good. Sensitivity analysis showed that the heterogeneity originated from the study of Nestler [24]. It was the only study in which the number of PPC cases was greater in the RM group than the control group. This may have been due to errors caused by the small samples, as only 25 patients per group were analyzed. After excluding this study, the results showed that a recruited pressure greater than 40 also reduced PPCs compared to the control group.

The PEEP should be manipulated following RMs to keep the alveoli open. Karsten et al. [39] demonstrated that the combination of the RM and PEEP guaranteed homogeneity in the local ventilation during laparoscopic surgery and enhanced the oxygenation and lung compliance based on electrical impedance tomography (EIT). In an observational study of 10,978 patients, Myrthe et al. [40] noted that mechanical ventilation combined with PEEP at 5–10 cm H_2_O was associated with fewer postoperative respiratory complications and shorter hospital stays in major abdominal surgery. Our subgroup analysis revealed that the incidence of PPCs was lower in the RM group, regardless of whether PEEP was performed in the control group. The protective effect of the RM coupled with PEEP was more apparent than that with ZEEP. This suggested that neither PEEP nor the RM alone was fully effective and that their combination was necessary to maximize the benefits. 

We analyzed two indices of pulmonary function: the static lung compliance and driving pressure. The results showed that RMs improved static lung compliance while decreasing the DP. This may be the mechanism by which RMs reduce PPCs. It has been shown that, among patients undergoing mechanical ventilation during general anesthesia, an increased DP is associated with more PPCs, and a lower DP may be lung protective [40]. Christopher et al. [6] also noted that the pulmonary compliance and driving pressures should be examined after RMs to assess the effects. We evaluated the oxygenation indices of patients during surgery and in the PACU. The fact that the RM improves intraoperative OIs has been confirmed by most studies. The RM needs to be maintained with a ventilator. There is a risk of the alveoli re-collapsing after detachment from the circuit. However, our results suggested that the RM was equally beneficial for improving oxygenation in the PACU. 

The hemodynamic stability during the RM is noteworthy. We evaluated two hemodynamic parameters: the MAP and HR. There was no significant difference between the control and RM groups. However, we cannot assume, on this basis, that the RM does not have any impact on the circulatory system. The point at which the data were recorded varied widely between studies, with some being recorded 60 min after pneumoperitoneum and others before the end of surgery. The parameters when carrying out the RM were not recorded. In fact, the increased transpulmonary pressure (TP) during RMs causes elevations of the central venous pressure (CVP), pulmonary vascular resistance index (PVRI) and pulmonary artery pressure (PAP), which raises the preload and afterload of the right ventricle, resulting in a transient decrease in the right and left ventricular ejection fraction (R/LVEF) during RMs. Celebi et al. [41] showed that the effect of the RM on the right ventricle was temporary and that the hemodynamics returned to normal with the release of the high airway pressure. Reis et al. [42] also demonstrated that the right ventricular work increases only during the first 2 min after intervention.

There are some limitations of this meta-analysis that need to be taken into account. First, the diagnostic criteria for PPCs varied among studies. Some studies reported the incidence of PPCs at 24 h postoperatively, while others reported PPCs at 5 or even 7 days following surgery. The PPCs were well defined in the high-quality studies but not explicitly stated in others. These factors may affect the accuracy of the conclusions. Second, the measurements for continuous data were conducted at different time points (e.g., 40, 50 or 60 min after pneumoperitoneum). Third, quantitative hemodynamic analysis is inadequate, and the safety of the RM remains to be further clarified. Fourth, the majority of the patients included in the study had normal cardiopulmonary function, so our conclusions may not be applicable to patients with severe cardiac or pulmonary disease.

## 5. Conclusions

Our systematic review and meta-analysis have demonstrated that the recruitment maneuver reduces postoperative pulmonary complications and improves respiratory mechanics and oxygenation in patients undergoing laparoscopic abdominal surgery. More data are needed to elucidate the effect of recruitment maneuver on the circulatory system. In general, the use of recruitment maneuver during mechanical ventilation maybe beneficial. Moreover, the long-term outcome parameters for the recruitment maneuver in patients and how to choose the optimal recruitment maneuver according to patient characteristics remain to be further explored.

## Figures and Tables

**Table 1:** Search strategies.

String	Condition	Search
#1	-	((“Abdomen”[Mesh]) OR “Laparoscopy”[Mesh]) OR “Hand-Assisted Laparoscopy”[Mesh]
#2	OR	((((((((((((((((((((((((((((((((((Laparoscop*[Title/Abstract]) OR (Celioscop*[Title/Abstract])) OR (Peritoneoscop*[Title/Abstract])) OR (Surgical Procedures, Laparoscopic[Title/Abstract])) OR (Laparoscopic Surgical Procedure[Title/Abstract])) OR (Procedure, Laparoscopic Surgical[Title/Abstract])) OR (Procedures, Laparoscopic Surgical[Title/Abstract])) OR (Surgery, Laparoscopic[Title/Abstract])) OR (Laparoscopic Surgical Procedures[Title/Abstract])) OR (Laparoscopic Surgery[Title/Abstract])) OR (Laparoscopic Surgeries[Title/Abstract])) OR (Surgeries, Laparoscopic[Title/Abstract])) OR (Laparoscopic Assisted Surgery[Title/Abstract])) OR (Laparoscopic Assisted Surgeries[Title/Abstract])) OR (Surgeries, Laparoscopic Assisted[Title/Abstract])) OR (Surgery, Laparoscopic Assisted[Title/Abstract])) OR (Surgical Procedure, Laparoscopic[Title/Abstract])) OR (Hand Assisted Laparoscop*[Title/Abstract])) OR (Laparoscopies, Hand-Assisted[Title/Abstract])) OR (Laparoscopy, Hand-Assisted[Title/Abstract])) OR (Hand-Assisted Laparoscopic Surgery[Title/Abstract])) OR (Hand Assisted Laparoscopic Surgery[Title/Abstract])) OR (Hand-Assisted Laparoscopic Surgeries[Title/Abstract])) OR (Laparoscopic Surgeries, Hand-Assisted[Title/Abstract])) OR (Laparoscopic Surgery, Hand-Assisted[Title/Abstract])) OR (Surgeries, Hand-Assisted Laparoscopic[Title/Abstract])) OR (Surgery, Hand-Assisted Laparoscopic[Title/Abstract])) OR (Hand-Assisted Laparoscopic Surgical Procedures[Title/Abstract])) OR (Hand Assisted Laparoscopic Surgical Procedures[Title/Abstract])) OR (abdomen[Title/Abstract])) OR (abdominal[Title/Abstract])) OR (belly[Title/Abstract])) OR (stomach[Title/Abstract])) OR (tummy[Title/Abstract])) OR (midriff[Title/Abstract])
#3	AND	((((((((recruitment maneuver [Title/Abstract]) OR (recruitment maneuver*[Title/Abstract])) OR (RM[Title/Abstract])) OR (open lung [Title/Abstract])) OR (open-lung [Title/Abstract])) OR (protected ventilation [Title/Abstract])) OR (protective ventilation [Title/Abstract])) OR (protected mechanical ventilation [Title/Abstract])) OR (protective mechanical ventilation [Title/Abstract])
#4	AND	(((randomized controlled trial [Publication Type]) OR (randomized [Title/Abstract])) OR (placebo [Title/Abstract])) OR (trial*[Title/Abstract])
#5	AND	(“1980/01/01”[Date—Publication]: “2021/12/31”[Date—Publication])
#6	-	[#1 OR #2] AND #3 AND #4 AND #5

Mesh: Medical Subject Headings; *: truncation function; [Title/Abstract]: search field; [Publication Type]: search field; [Date—Publication]: search field; OR: Boolean logic operator; AND: Boolean logic operator.

**Table 2:** Study characteristics.

Study	Surgery	No. of Patients (Male/Female)	Age, Years Mean (SD)	ASA Class	BMI, kg/m^2^, Mean (SD)	Tidal Volume (ml/kg)	Recruitment Maneuver	PEEP (cmH_2_O)
Almarakbi, 2009	laparoscopic gastric banding	C:15 (8/7) RM1: 15 (9/6) RM2:15 (7/8) RM3:15 (8/7)	C: 38 (3) RM1: 38 (3) RM2: 38 (3) RM3: 38 (4)	II	C: 33 (2) RM1: 33 (1) RM2: 34 (1) RM3: 33 (1)	C:10 RM1:10 RM2:10 RM3:10	sustained inspiratory pressure of 40 cm H_2_O for 15 s; performed 10 min after pneumoperitoneum, before surgery	C:10 RM1:0 RM2:10 RM3:10
Bluth, 2019	general anesthesia surgery	RM:740 (221/519) C:727 (221/506)	RM:48.6 (13.8) C:48.9 (13.3)	I–IV	> 35	RM:7 C:7	The tidal volume was increased 4 mL/kg until Pplat reaches 40–50 cmH_2_O,3 breaths for 40–50 cmH_2_O; performed after induction of anesthesia, after any disconnection from the mechanical ventilator, every 1 h during surgery, and before extubation	RM:12 C:4
Choi, 2017	RARP	RM:26 (26/0) C:25 (25/0)	RM:67.6 (4.3) C:66.6 (4.3)	I–II	RM:24.5 (2) C:24.5 (2.1)	RM:6–8 C:6–8	staircase PEEP(4–16 cmH_2_O), Ppeak < 35 cmH_2_O; performed after intubation	RM:5 C:5
Ferrando, 2018	abdominal surgery	RM1:241 (141/100) RM2:237 (157/80) C1:243 (163/80) C2:244 (154/90)	RM1:64.3 (13) RM2:64.7 (13.2) C1:66.5 (11.4) C2:64.8 (12.9)	I–IV	RM1:26 (4) RM2:26.2 (4) C1:25.8 (3.7) C2:26.1 (3.9)	RM1:8 RM2:8 C1:8 C2:8	step-wise RM until Paw reached 40 cm H_2_O + PEEP titration trial; performed after intubation, every 40 min during surgery	RM1:iPEEP RM2:iPEEP C1:5 C2:5
Fuiter, 2013	abdominal surgery	RM:41 (200) C:44 (200)	RM:61.6 (11) C:63.4 (10)	N	RM:24.8 (3.8) C:24.7 (3.8)	RM:6.4 (0.8) C:11.1 (1.1)	CPAP 30 cm H_2_O for 30 s; performed after pneumoperitoneum, every 0.5 h during surgery	RM:6–8 C: 0
Li, 2021	laparoscopic colorectal cancer resection	RM:130 (102/28) C:130 (98/32)	RM:69.7 (5.8) C:70.8 (5.8)	II–III	RM:23 (2.7) C:22.3 (2.8)	RM:6–8 C:6–8	PEEP= 12 cm H_2_O, tidal volumes are increased in steps of 4 mL/kg PBW until a plateau pressure of 30–35 cm H_2_O, 3 breaths for 30–35 cm H_2_O; performed after induction of anesthesia, every 0.5 h during surgery	RM:6–8 C:0
Liu, 2019	laparoscopic gastric cancer radical surgery	RM:58 (26/32) C:57 (26/31)	RM:63.2 (8.31) C:66.13 (9.12)	I–III	RM:22.45 (2.1) C:23.27 (2.95)	RM:7 C:10	CPAP and applying 30 cm H_2_O PEEP for 30 s; followed by a decremental PEEP titration procedure	RM:iPEEP C:0
Liu, 2020	laparoscopic total hysterectomy	RM:44 (0/44) C:43 (0/43)	RM:51.08 (8.86) C:50.32 (9.83)	I–III	RM:23.31 (3.98) C:22.58 (3.05)	RM:7 C:9	30 cm H_2_O PEEP for 30 s followed by a decremental PEEP titration procedure; immediately after induction of anesthesia and orotracheal intubation	RM:iPEEP C:0
Nestler, 2017	elective laparoscopic surgery	RM:25 (8/17) C:25 (8/17)	RM:44.9 (11.14) C:46.2 (12.57)	N	RM:48.3 (7.1) C:53.8 (8.2)	RM:8 C:8	Ppeak < 50 H_2_O, PEEP 30 cm H_2_O, RR 6 bpm, for 10 cycles; an RM followed by a decremental PEEP titration, additional RM was performed before extubating	RM:iPEEP [18(4.27)] C:5
Nguyen, 2021	laparoscopic abdominal surgery	RM:31 (9/22) C:31 (13/18)	RM:59 (9) C:55 (12)	II–III	RM:21 (2) C:21 (3)	RM:7 C:10	staircase PEEP (10,15,20 cm H_2_O), PIP < 50 cm H_2_O; right after intubation, 30 min after CO_2_ insufflation, then every hour, and finally before extubating	RM:10 C:0
Paula, 2011	bariatric surgery by video-laparoscopy	RM:15 (4/11) C:15 (5/10)	RM:42.1 (14.5) C:37.2 (12.2)	N	RM:35.2 (5.5) C:35.4 (5.5)	RM:11.5 (2.36) C:10.8 (1.3)	PEEP of 30 cm H_2_O and inspiratory plateau pressure of 15 cm H_2_O above PEEP for 2 min	RM: 5.7 (0.9) C: 5.4 (0.91)
Mathilde, 2021	laparoscopic bariatric surgery	RM:115 (19/96) C:113 (26/87)	RM:38.8 (15) C:39.4 (17.6)	I–III	41 (4.5)	RM:6–8 C:6–8	maintaining the airway pressure at 30 cm H_2_O for 30 s; performed after intubation and every 30 min for the all duration of anesthesia	RM:5–10 C:5–10
Sheree, 2020	LSG	RM:23 (11/12) C1:23 (10/13) C2:23 (12/11)	RM:29.8 (8.98) C1:29.7 (8.2) C2:29.7 (9.3)	II–III	RM:39.3 (1.9) C1:39.5 (2.5) C2:39 (2.68)	RM:6–8 C1:6–8 C2:6–8	airway pressure 40 cm H_2_O for 40 s; performed post-induction of anesthesia, 2 min after completion of pneumoperitoneum, 2 min after placing the patient in Trendelenburg position, and finally 2 min after exsufflation of pneumoperitoneum	RM:5 C1:5 C2:0
Sowoon, 2016	RALP	RM:30 (30/0) C:30 (30/0)	RM:63 (6) C:62 (6)	N	N	RM:6 C:6	CPAP of 40 cm H_2_O for 40 s; 15 min after Trendelenburg position	RM:15 C:15
Wei, 2018	LSG	RM1:12 (6/6) RM2:11 (5/6) C:12 (5/7)	RM1:39 (10.48) RM2:33 (10.49) C:37 (14)	II–III	RM1:43 (6) RM2:48 (8) C:45 (6)	RM1:8 RM2:8 C:8	staircase PEEP (5–15 cm H_2_O), Ppeak < 40; performed immediately after the inflation of pneumoperitoneum, repeated every 30 min during the procedure, the last RM followed the deflation of pneumoperitoneum.	RM1:8 RM2:0 C:0
Yang, 2021	laparoscopic surgery for colorectal cancer	RM:20 (16/4) C:20 (14/6)	RM:66.4 (4.6) C:69.5 (6.2)	II–III	RM:22.3 (2.2) C:22.7 (2.3)	RM:6–8 C:6–8	gradual rise in airway pressure under ultrasound guidance from 10 cm H_2_O by 5 cmH_2_O increments, until no collapsed lung areas were visible on the sonogram, the pressure was maintained for 40 s, Ppeak < 40 cm H_2_O; performed at the end of the surgery	RM:4 C:4
Youn, 2020	laparoscopic low anterior resection for colorectal cancer	RM:30 (15/15) C:32 (19/13)	RM:74 (5) C:76 (7)	I–II	N	RM:6 C:6	staircase PEEP (10, 15, 20 cm H_2_O), 3 breaths for each PEEP, Ppeak < 40 cm H_2_O; immediately before and after CO_2_ pneumoperitoneum	RM:5 C:5

SD: standard deviation; ASA: American Society of Anesthesiologists; BMI: body mass index; PEEP: positive end-expiratory pressure; C: control; RM: recruitment maneuver; Pplat: plateau pressure; RARP: robot-assisted laparoscopic radical prostatectomy; Ppeak: peak airway pressure; iPEEP: individualized positive end-expiratory pressure; CPAP: continuous positive airway pressure; PBW: predicted body weight; N: not reported; PIP: peak inspiratory pressure; CO_2_: carbon dioxide; RR: respiratory rate; bpm: breaths per minute; RALP: robot-assisted laparoscopic prostatectomy; LSG: laparoscopic sleeve gastrectomy.

**Table 3:** Quality of evidence by GRADE.

Quality Assessment							No of Patients		Effect		Quality	Importance
No of Studies	Design	Risk of Bias	Inconsistency	Indirectness	Imprecision	Other Considerations	RM	Control	Relative (95% CI)	Absolute		
**PPC**												
17	randomized trials	no serious risk of bias	no serious inconsistency	no serious indirectness	no serious imprecision	none	314/1746 (18%)	448/1734 (25.8%)	RR 0.7 (0.62 to 0.79)	78 fewer per 1000 (from 54 fewer to 98 fewer)	ÅÅÅÅ HIGH	CRITICAL
								25.6%		77 fewer per 1000 (from 54 fewer to 97 fewer)		
**PPC-BMI ≥ 35**												
6	randomized trials	no serious risk of bias	no serious inconsistency	no serious indirectness	no serious imprecision	none	137/928 (14.8%)	172/915 (18.8%)	RR 0.79 (0.64 to 0.96)	39 fewer per 1000 (from 8 fewer to 68 fewer)	ÅÅÅÅ HIGH	CRITICAL
								6.4%		13 fewer per 1000 (from 3 fewer to 23 fewer)		
**PPC-BMI < 35**												
8	randomized trials	no serious risk of bias	no serious inconsistency	no serious indirectness	no serious imprecision	none	163/743 (21.9%)	256/742 (34.5%)	RR 0.64 (0.54 to 0.75)	124 fewer per 1000 (from 86 fewer to 159 fewer)	ÅÅÅÅ HIGH	CRITICAL
								32.5%		117 fewer per 1000 (from 81 fewer to 149 fewer)		
**PPC-Age < 65**												
6	randomized trials	no serious risk of bias	no serious inconsistency	no serious indirectness	no serious imprecision	none	135/918 (14.7%)	172/905 (19%)	RR 0.77 (0.63 to 0.95)	44 fewer per 1000 (from 10 fewer to 70 fewer)	ÅÅÅÅ HIGH	CRITICAL
								6.4%		15 fewer per 1000 (from 3 fewer to 24 fewer)		
**PPC- Single RM**												
5	randomized trials	no serious risk of bias	no serious inconsistency	no serious indirectness	no serious imprecision	strong association 1	13/127 (10.2%)	36/125 (28.8%)	RR 0.36 (0.21 to 0.64)	184 fewer per 1000 (from 104 fewer to 228 fewer)	ÅÅÅÅ HIGH	CRITICAL
								25.6%		164 fewer per 1000 (from 92 fewer to 202 fewer)		
**PPC-Repeated RM**												
12	randomized trials	no serious risk of bias	no serious inconsistency	no serious indirectness	no serious imprecision	none	301/1619 (18.6%)	412/1609 (25.6%)	RR 0.73 (0.64 to 0.83)	69 fewer per 1000 (from 44 fewer to 92 fewer)	ÅÅÅÅ HIGH	CRITICAL
								24.5%		66 fewer per 1000 (from 42 fewer to 88 fewer)		
**PPC- Stepwise RM**												
8	randomized trials	no serious risk of bias	no serious inconsistency	no serious indirectness	no serious imprecision	none	277/1223 (22.6%)	357/1213 (29.4%)	RR 0.77 (0.68 to 0.88)	68 fewer per 1000 (from 35 fewer to 94 fewer)	ÅÅÅÅ HIGH	CRITICAL
								34.3%		79 fewer per 1000 (from 41 fewer to 110 fewer)		
**PPC-Sustained RM**												
9	randomized trials	no serious risk of bias	no serious inconsistency	no serious indirectness	no serious imprecision	strong association 1	37/523 (7.1%)	91/521 (17.5%)	RR 0.41 (0.29 to 0.58)	103 fewer per 1000 (from 73 fewer to 124 fewer)	ÅÅÅÅ HIGH	CRITICAL
								5.3%		31 fewer per 1000 (from 22 fewer to 38 fewer)		
**PPC-Recruited pressure ≥ 40**												
5	randomized trials	no serious risk of bias	no serious inconsistency	no serious indirectness	no serious imprecision	none	8/108 (7.4%)	17/108 (15.7%)	RR 0.5 (0.24 to 1.04)	79 fewer per 1000 (from 120 fewer to 6 more)	ÅÅÅÅ HIGH	CRITICAL
								4.4%		22 fewer per 1000 (from 33 fewer to 2 more)		
**PPC-Recruited pressure < 40**												
4	randomized trials	no serious risk of bias	no serious inconsistency	no serious indirectness	no serious imprecision	strong association 1	28/327 (8.6%)	69/325 (21.2%)	RR 0.41 (0.27 to 0.61)	125 fewer per 1000 (from 83 fewer to 155 fewer)	ÅÅÅÅ HIGH	CRITICAL
								15.4%		91 fewer per 1000 (from 60 fewer to 112 fewer)		
**PPC-Compare to ZEEP**												
7	randomized trials	no serious risk of bias	no serious inconsistency	no serious indirectness	no serious imprecision	strong association 1	60/498 (12%)	125/496 (25.2%)	RR 0.48 (0.37 to 0.64)	131 fewer per 1000 (from 91 fewer to 159 fewer)	ÅÅÅÅ HIGH	CRITICAL
								25.6%		133 fewer per 1000 (from 92 fewer to 161 fewer)		
**PPC-Compare to PEEP**												
10	randomized trials	no serious risk of bias	no serious inconsistency	no serious indirectness	no serious imprecision	none	254/1248 (20.4%)	323/1238 (26.1%)	RR 0.78 (0.68 to 0.9)	57 fewer per 1000 (from 26 fewer to 83 fewer)	ÅÅÅÅ HIGH	CRITICAL
								27.7%		61 fewer per 1000 (from 28 fewer to 89 fewer)		
**Static lung compliance**												
7	randomized trials	no serious risk of bias	no serious inconsistency	no serious indirectness	serious 2	none	315	313	-	MD 10.42 higher (6.13 to 14.71 higher)	ÅÅÅO MODERATE	CRITICAL
**Driving pressure**												
7	randomized trials	no serious risk of bias	no serious inconsistency	no serious indirectness	serious 2	none	1308	1295	-	MD 3.96 lower (5.97 to 1.95 lower)	ÅÅÅO MODERATE	CRITICAL
**Intraoperative oxygenation index**												
11	randomized trials	no serious risk of bias	no serious inconsistency	no serious indirectness	serious 2	none	641	644	-	MD 53.54 higher (21.77 to 85.31 higher)	ÅÅÅO MODERATE	CRITICAL
**Postoperative oxygenation index**												
7	randomized trials	no serious risk of bias	no serious inconsistency	no serious indirectness	serious 2	none	934	919	-	MD 59.4 higher (39.1 to 79.69 higher)	ÅÅÅO MODERATE	CRITICAL
**Mean arterial pressure**												
7	randomized trials	no serious risk of bias	no serious inconsistency	no serious indirectness	no serious imprecision	none	1092	1085	-	MD 0.16 lower (1.35 lower to 1.03 higher)	ÅÅÅÅ HIGH	CRITICAL
**Heart rate**												
6	randomized trials	no serious risk of bias	no serious inconsistency	no serious indirectness	no serious imprecision	none	851	841	-	MD 1.1 lower (2.29 lower to 0.1 higher)	ÅÅÅÅ HIGH	CRITICAL

Value of 1 RR < 0.5 suggests a significant effect. Value of 2 I^2^ > 50% indicated high heterogeneity.

## Data Availability

No new data were created or analyzed in this study. Data sharing is not applicable to this article.
